# How Should I Submit to *mSphere*: Traditional, Expedited, or mSphereDirect?

**DOI:** 10.1128/mSphere.00419-17

**Published:** 2017-10-11

**Authors:** Michael J. Imperiale

**Affiliations:** Department of Microbiology and Immunology, University of Michigan, Ann Arbor, Michigan, USA

## EDITORIAL

As we approach the end of our second year serving the microbial science community, we have been examining our processes as well as listening to the formal and informal feedback that we have received from the community. I take this opportunity to try to clarify some misconceptions and tell you about some changes to our processes, with the goal of helping you decide the best way to submit your exciting work to *mSphere*.

Since the beginning of 2017, *mSphere* has offered the mSphereDirect pathway as an alternative to the traditional peer review mechanism, which we still offer (1). mSphereDirect puts authors in control of the review process, allowing them to solicit their own reviews from qualified reviewers, respond to those reviews, and submit a revised manuscript to the journal along with the reviews for a rapid "yes" or "no" decision. Overall, the *mSphere* editorial team is pleased with the way this experiment is progressing, and we have already published a number of excellent manuscripts.

We have, however, noticed a couple of parts of the system that require some clarification, to better describe the roles of the authors and the reviewers and to assist the Senior Editors in the decision-making process, particularly as it relates to the significance of the work. Specifically, we are implementing new guidance about our expectations from authors and reviewers and are slightly changing the review form. The overarching goal is to maintain our commitment to publishing only those papers that are of high importance to those working in the microbial sciences, whether submitted through the regular or the mSphereDirect pathway.

We are providing additional guidance to authors about how to undertake the review process, as some authors have been unsure about the best way to approach potential reviewers or to handle the iterative process of responding to the reviews. We are also asking reviewers to comment more specifically about the importance of the work and to assert on the review form that they have read the revised manuscript and are satisfied with the author responses to the original reviews. This means that reviewers will need to send the review form to the authors and that the authors will subsequently need to return the review form, along with the revised manuscript and the rebuttal, to allow the reviewers to attest to having seen these documents. We are also asking authors to upload a “tracked-changes” version of the revised manuscript.

The details of these procedural changes can be found in the *mSphere* and mSphereDirect Instructions to Authors (http://msphere.asm.org/sites/default/files/additional-assets/mSph-ITA-July-2017.pdf and http://msphere.asm.org/sites/default/files/additional-assets/mSphereDirect-ITA.pdf) and FAQ pages (http://msphere.asm.org/content/faq and http://msphere.asm.org/content/mspheredirect-author-faqs). In addition, we have uploaded what we believe to be a great example of how the process should take place (http://msphere.asm.org/sites/default/files/additional-assets/mSDSample.pdf). We are grateful to Karla Satchell and her coauthors, as well as to Carmen Amaro and Jonathon Audia, the external solicited reviewers, for doing such a wonderful job and for graciously allowing us to post their documents relating to the Satchell laboratory’s report on *Vibrio vulnificus* virulence (2).

I also remind authors that *mSphere* offers you the possibility of submitting manuscripts that have been peer reviewed and rejected by other high-quality journals for expedited review through our normal submission pathway. If you think that you can address the reviews in a revised manuscript, please contact me or the appropriate Senior Editor (based on expertise) via e-mail to see whether we would consider the expedited option.

We thank all the authors and reviewers who have contributed to mSphereDirect so far. We look forward to your submissions, and we are also eager to hear your feedback as we move into our third year.
